# 1,4-Bis(thio­phen-2-yl)butane-1,4-dione

**DOI:** 10.1107/S1600536812005338

**Published:** 2012-02-10

**Authors:** Wei-Ting Guo, Zhi-Min Miao, Yun-Long Wang

**Affiliations:** a Gout Laboratory, The Affiliated Hospital of Medical College Qingdao University, 16 Jiangsu Road, Qingdao, Shandong 266003, People’s Republic of China

## Abstract

In the centrosymmetric title compound, C_12_H_10_O_2_S_2_, the alkyl chains adopt a fully extended all-*trans* conformation with respect to the C(thio­phene)—C bond. The non-H atoms of the mol­ecule are nearly planar, with a maximum deviation of 0.063 (2) Å from the mean plane of the constituent atoms. In the crystal, symmetry-related mol­ecules are linked *via* pairs of C—H⋯π contacts [H–centroid distances of the thio­phene units = 2.79 (9) and 2.82 (4) Å], in turn inter­digitating with each other along the *bc* plane, thus leading to an inter­woven two-dimensional network.

## Related literature

For related structures, see: Becerra *et al.* (2010 ▶); Liu *et al.* (2008 ▶); Nair, Devipriya & Eringathodi (2007 ▶); Nair, Vellalath *et al.* (2007 ▶); Bushueva *et al.* (2010 ▶). For background information on applications, see: Atalar *et al.* (2009 ▶); Chen *et al.* (2009 ▶); Charati *et al.* (2008 ▶); Cao *et al.* (2008 ▶); Wu *et al.* (2008 ▶). For the synthetic procedure, see: Schweiger *et al.* (2000 ▶). For bond lengths, see: Allen *et al.* (1987 ▶). For related C—H⋯π hydrogen bonds, see: Hu *et al.* (2008 ▶); Ishihara *et al.* (2007 ▶); Jennings *et al.* (2001 ▶).
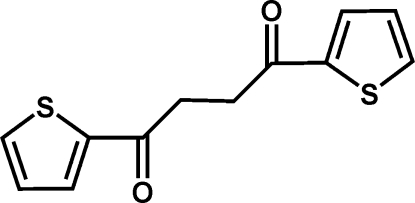


## Experimental

### 

#### Crystal data

C_12_H_10_O_2_S_2_
*M* *_r_* = 250.34
Monoclinic, 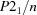

*a* = 5.6345 (3) Å
*b* = 6.2244 (3) Å
*c* = 16.3779 (9) Å
β = 92.902 (4)°
*V* = 573.66 (5) Å^3^
*Z* = 2
Mo *K*α radiation
μ = 0.44 mm^−1^
*T* = 296 K
0.20 × 0.15 × 0.10 mm

#### Data collection

Bruker SMART CCD area-detector diffractometer
Absorption correction: multi-scan (*SADABS*; Sheldrick, 1996 ▶) *T*_min_ = 0.957, *T*_max_ = 0.978
2000 measured reflections
1023 independent reflections
838 reflections with *I* > 2σ(*I*)
*R*_int_ = 0.021

#### Refinement

*R*[*F* ^2^ > 2σ(*F* ^2^)] = 0.062
*wR*(*F* ^2^) = 0.200
*S* = 1.09
1023 reflections
73 parameters
H-atom parameters constrained
Δρ_max_ = 0.58 e Å^−3^
Δρ_min_ = −0.35 e Å^−3^

### 

Data collection: *SMART* (Bruker, 2007 ▶); cell refinement: *SAINT* (Bruker, 2007 ▶); data reduction: *SAINT*; program(s) used to solve structure: *SHELXS97* (Sheldrick, 2008 ▶); program(s) used to refine structure: *SHELXL97* (Sheldrick, 2008 ▶); molecular graphics: *SHELXTL* (Sheldrick, 2008 ▶); software used to prepare material for publication: *SHELXTL*.

## Supplementary Material

Crystal structure: contains datablock(s) I, global. DOI: 10.1107/S1600536812005338/zj2057sup1.cif

Structure factors: contains datablock(s) I. DOI: 10.1107/S1600536812005338/zj2057Isup2.hkl

Supplementary material file. DOI: 10.1107/S1600536812005338/zj2057Isup3.cml

Additional supplementary materials:  crystallographic information; 3D view; checkCIF report

## Figures and Tables

**Table 1:** Hydrogen-bond geometry (Å, °) *Cg*1 is the centroid of the C1–C4/S1 ring.

*D*—H⋯*A*	*D*—H	H⋯*A*	*D*⋯*A*	*D*—H⋯*A*
C1—H1⋯*Cg*1^i^	0.93	2.79 (9)	3.610 (5)	146
C6—H6*B*⋯*Cg*1^ii^	0.97	2.82 (4)	3.637 (4)	143

Symmetry codes: (i) 
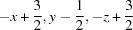
; (ii) 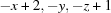.

**Fig. 1.**
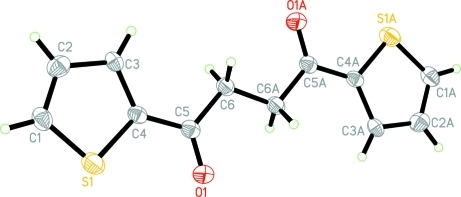
The molecular structure of the title compound, with displacement ellipsoids at the 30% probability level (Symmetry code: (A) 2-x, 1-y, 1-z).

**Fig. 2.**
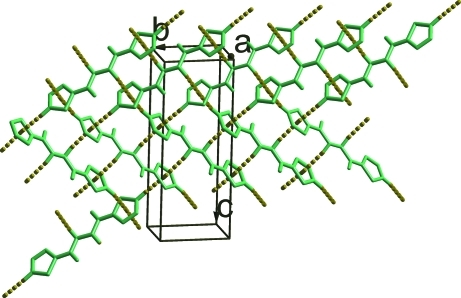
The C–H···π stacking interactions (dashed lines) in the structure of the title compound viewing along bc plane, H atoms have been omitted for clarity, except for those involved in C–H···π stacking interactions.

## supplementary crystallographic information

### Comment

Electroactive and photoactive thiophene-based molecules, oligomers, and polymers (Atalar *et al.*, 2009; Chen *et al.*, 2009; Charati *et al.*, 2008; Becerra *et al.*, 2010; Liu *et al.*, 2008; Nair, Devipriya & Eringathodi, 2007; Nair, Vellalath *et al.*, 2007; Bushueva *et al.*; 2010) are important for advanced technological applications, including display technologies, field-effect transistors, solar cells, sensors, nonlinear optics, molecular wires, and diodes (Cao *et al.*, 2008; Wu *et al.*, 2008). Thiophenes and its derivatives are originally applied to synthesize thiophene-based compounds, such as, oligomers and polythiophenes due to easy electropolymerization of thiophenes to produce stable, electrically conductive polymeric films. Thiophenes and its derivatives can also be widely used as building blocks in many agrochemicals and pharmaceuticals. To investigate the relationship between structure and pharmacological activity, the title compound was synthesized and its structure was determined by X-ray diffraction. As shown in figure 1, the centrosymmetric title compound lies across a crystallographic inversion centre which is situated at the midpoint of the C6–C6A (1.512 (8) Å, symmetry code: (A) 2-x, 1-y, 1-z) bond. The alkyl chains attached thiophenes adopt a fully extended all-*trans* conformation with respect to the C_thiophene_–C bond. The non-H atoms of the molecule is nearly planar with O1 and O1A maximum deviation of 0.063 (2) Å from the mean plane of its constituting atoms. The C1–S1 and C4–S1 bond length of 1.678 (5) and 1.697 (4) Å, respectively, correspond to typical single C–S bonds. Whereas the C1–O1 (1.215 (5) Å) bond length indicate the presence of a typical double C═O bond (Allen *et al.*, 1987). It is well-established that weak bonding interactions between entities, such as hydrogen-bonding and π-stacking, are important supramolecular forces, which can be used to govern the process of molecular recognition and selfassembly (Hu *et al.*, 2008; Ishihara *et al.*, 2007; Jennings *et al.*, 2001). In the crystal structure, it is noteworthy that symmetry-related molecules, in turn, are linked *via* pairs of intermolecular C—H···π constacts [H–centroid distance of the thiophene units = 2.79 (9) - 2.82 (4) Å], and interdigitated with each other along *bc* plane, thus leading to an interwoven two dimensional network.

### Experimental

Reagents and solvents were of commercially available quality. The title compound was synthesized according to the method of Schweiger *et al.* 2000. To a suspension of AlCl_3_ (16 g, 0.12 mol) in CH_2_Cl_2_ (15 ml) a solution of thiophene (10 ml, 0.12 mol) and succinyl chloride (6 ml, 0.05 mol) in CH_2_Cl_2_ was added dropwise. The red mixture was stirred at r.t. for 18 h. This was then quenched with ice and conc. HCl (5 ml). After intensive stirring for 2 h the dark green organic phase was separated, washed with 2 M HCl, H_2_O NaHCO_3_ solution and dried over MgSO_4_. After evaporation of the solvent a blue-green solid remained, which was suspended in ethanol. Filtration and washing with ethanol and diethyl ether provided a green solid. Column chromatography (SiO_2_, CH_2_Cl_2_-hexane (1 : 1)) obtains (I) as a white solid 6.74 g (26.87 mmol; 54%). Single crystals suitable for X-ray diffraction were prepared by slow evaporation of a solution of the crude (5 g) in a mixture of water/ethanol (20 ml, 1:1, *v*/*v*) at room temperature.

### Refinement

All H atoms were placed in idealized positions (C—H = 0.93–0.97 Å) and refined as riding atoms with *U*_iso_(H) = 1.2*U*_eq_(C).

### Crystal data

**Table:**

C_12_H_10_O_2_S_2_	*F*(000) = 260
*M**_r_* = 250.34	*D*_x_ = 1.449 Mg m^−^^3^
Monoclinic, *P*2_1_/*n*	Mo *K*α radiation, λ = 0.71073 Å
Hall symbol: -P 2yn	Cell parameters from 1252 reflections
*a* = 5.6345 (3) Å	θ = 2.6–22.6°
*b* = 6.2244 (3) Å	µ = 0.44 mm^−^^1^
*c* = 16.3779 (9) Å	*T* = 296 K
β = 92.902 (4)°	Bolck, yellow
*V* = 573.66 (5) Å^3^	0.20 × 0.15 × 0.10 mm
*Z* = 2	

### Data collection

**Table:**

Bruker SMART CCD area-detector diffractometer	1023 independent reflections
Radiation source: fine-focus sealed tube	838 reflections with *I* > 2σ(*I*)
Graphite monochromator	*R*_int_ = 0.021
φ and ω scans	θ_max_ = 25.1°, θ_min_ = 3.5°
Absorption correction: multi-scan (*SADABS*; Sheldrick, 1996)	*h* = −5→6
*T*_min_ = 0.957, *T*_max_ = 0.978	*k* = −7→6
2000 measured reflections	*l* = −19→19

### Refinement

**Table:**

Refinement on *F*^2^	Primary atom site location: structure-invariant direct methods
Least-squares matrix: full	Secondary atom site location: difference Fourier map
*R*[*F*^2^ > 2σ(*F*^2^)] = 0.062	Hydrogen site location: inferred from neighbouring sites
*wR*(*F*^2^) = 0.200	H-atom parameters constrained
*S* = 1.09	*w* = 1/[σ^2^(*F*_o_^2^) + (0.105*P*)^2^ + 0.7893*P*] where *P* = (*F*_o_^2^ + 2*F*_c_^2^)/3
1023 reflections	(Δ/σ)_max_ < 0.001
73 parameters	Δρ_max_ = 0.58 e Å^−^^3^
0 restraints	Δρ_min_ = −0.35 e Å^−^^3^

### Special details

**Table:**

Geometry. All esds (except the esd in the dihedral angle between two l.s. planes) are estimated using the full covariance matrix. The cell esds are taken into account individually in the estimation of esds in distances, angles and torsion angles; correlations between esds in cell parameters are only used when they are defined by crystal symmetry. An approximate (isotropic) treatment of cell esds is used for estimating esds involving l.s. planes.
Refinement. Refinement of F^2^ against ALL reflections. The weighted R-factor wR and goodness of fit S are based on F^2^, conventional R-factors R are based on F, with F set to zero for negative F^2^. The threshold expression of F^2^ > 2sigma(F^2^) is used only for calculating R-factors(gt) etc. and is not relevant to the choice of reflections for refinement. R-factors based on F^2^ are statistically about twice as large as those based on F, and R- factors based on ALL data will be even larger.

### Fractional atomic coordinates and isotropic or equivalent isotropic displacement parameters (Å^2^)

**Table:**

	*x*	*y*	*z*	*U*_iso_*/*U*_eq_	
S1	1.1013 (2)	−0.0356 (2)	0.69663 (7)	0.0577 (5)	
C4	0.9593 (6)	0.0981 (6)	0.6181 (2)	0.0376 (9)	
C5	1.0715 (7)	0.2877 (6)	0.5837 (2)	0.0403 (9)	
C3	0.7295 (7)	0.0004 (6)	0.5931 (2)	0.0368 (9)	
H3	0.6239	0.0483	0.5516	0.044*	
C2	0.6983 (7)	−0.1823 (8)	0.6446 (3)	0.0537 (12)	
H2	0.5649	−0.2702	0.6398	0.064*	
C1	0.8824 (8)	−0.2171 (8)	0.7017 (3)	0.0515 (11)	
H1	0.8850	−0.3295	0.7392	0.062*	
O1	1.2671 (5)	0.3465 (5)	0.6091 (2)	0.0592 (9)	
C6	0.9351 (7)	0.4030 (7)	0.5154 (2)	0.0419 (10)	
H6A	0.7830	0.4485	0.5346	0.050*	
H6B	0.9043	0.3038	0.4705	0.050*	

### Atomic displacement parameters (Å^2^)

**Table:**

	*U*^11^	*U*^22^	*U*^33^	*U*^12^	*U*^13^	*U*^23^
S1	0.0511 (8)	0.0646 (9)	0.0569 (8)	0.0067 (5)	−0.0020 (5)	0.0135 (6)
C4	0.0371 (19)	0.040 (2)	0.0356 (19)	0.0092 (16)	0.0000 (15)	−0.0042 (17)
C5	0.041 (2)	0.040 (2)	0.040 (2)	0.0079 (17)	0.0006 (16)	−0.0056 (17)
C3	0.039 (2)	0.038 (2)	0.0348 (19)	0.0114 (16)	0.0097 (15)	0.0031 (16)
C2	0.042 (2)	0.058 (3)	0.062 (3)	−0.002 (2)	0.012 (2)	−0.005 (2)
C1	0.047 (2)	0.054 (3)	0.055 (2)	0.008 (2)	0.0129 (19)	0.016 (2)
O1	0.0523 (18)	0.057 (2)	0.067 (2)	−0.0076 (15)	−0.0175 (15)	0.0107 (17)
C6	0.045 (2)	0.039 (2)	0.041 (2)	0.0047 (18)	−0.0052 (16)	−0.0003 (18)

### Geometric parameters (Å, º)

**Table:**

S1—C1	1.678 (5)	C3—H3	0.9300
S1—C4	1.697 (4)	C2—C1	1.379 (6)
C4—C5	1.466 (6)	C2—H2	0.9300
C4—C3	1.470 (6)	C1—H1	0.9300
C5—O1	1.215 (5)	C6—C6^i^	1.512 (8)
C5—C6	1.506 (5)	C6—H6A	0.9700
C3—C2	1.431 (6)	C6—H6B	0.9700
			
C1—S1—C4	92.8 (2)	C1—C2—H2	122.8
C5—C4—C3	128.1 (3)	C3—C2—H2	122.8
C5—C4—S1	119.4 (3)	C2—C1—S1	112.9 (3)
C3—C4—S1	112.5 (3)	C2—C1—H1	123.5
O1—C5—C4	120.8 (4)	S1—C1—H1	123.5
O1—C5—C6	122.1 (4)	C5—C6—C6^i^	113.0 (4)
C4—C5—C6	117.1 (3)	C5—C6—H6A	109.0
C2—C3—C4	107.3 (3)	C6^i^—C6—H6A	109.0
C2—C3—H3	126.4	C5—C6—H6B	109.0
C4—C3—H3	126.4	C6^i^—C6—H6B	109.0
C1—C2—C3	114.5 (4)	H6A—C6—H6B	107.8
			
C1—S1—C4—C5	−179.8 (3)	S1—C4—C3—C2	−0.1 (4)
C1—S1—C4—C3	−0.1 (3)	C4—C3—C2—C1	0.3 (5)
C3—C4—C5—O1	−177.6 (4)	C3—C2—C1—S1	−0.3 (5)
S1—C4—C5—O1	2.0 (5)	C4—S1—C1—C2	0.2 (4)
C3—C4—C5—C6	1.7 (6)	O1—C5—C6—C6^i^	−1.1 (7)
S1—C4—C5—C6	−178.6 (3)	C4—C5—C6—C6^i^	179.6 (4)
C5—C4—C3—C2	179.6 (4)		

Symmetry code: (i) −*x*+2, −*y*+1, −*z*+1.

### Hydrogen-bond geometry (Å, º)

Cg1 is the centroid of the C1–C4/S1 or C1A–C4A/S1A ring.

**Table:**

*D*—H···*A*	*D*—H	H···*A*	*D*···*A*	*D*—H···*A*
C1—H1···*Cg*1^ii^	0.93	2.79 (9)	3.610 (5)	146
C6—H6*B*···*Cg*1^iii^	0.97	2.82 (4)	3.637 (4)	143

Symmetry codes: (ii) −*x*+3/2, *y*−1/2, −*z*+3/2; (iii) −*x*+2, −*y*, −*z*+1.
